# Basaloid squamous cell carcinoma clinically and radiologically masquerading as a head and neck paraganglioma: a case report and review of the literature

**DOI:** 10.1186/s13256-024-04601-4

**Published:** 2024-06-11

**Authors:** Pumudu Weerasekara, Nadeeka Chandrarathne, Geethika Jayaweera, Wasantha Rathnayake, Sunil Perera

**Affiliations:** 1 Faculty of Medicine, University of Colombo, Colombo, 00800 Sri Lanka; 2 Department of Community Medicine, Faculty of Medicine, University of Colombo, Colombo, 00800 Sri Lanka; 3 Department of Pathology, Asiri Central Hospital, Colombo, 01000 Sri Lanka; 4 Department of Oncology, National Cancer Institute, Maharagama, Sri Lanka; 5 Asiri Central Brain and Spine Neurosurgical Group, Asiri Central Hospital, Colombo, 01000 Sri Lanka

**Keywords:** Glomus jugulare, Head and neck paraganglioma, Basaloid squamous cell carcinoma, Case report

## Abstract

**Background:**

This paper reports the first case of basaloid squamous cell carcinoma clinically and radiologically masquerading as a head and neck paraganglioma.

**Case presentation:**

A 66-year-old Sinhalese male with unilateral hearing impairment and 7th–12th (excluding 11th) cranial nerve palsies was diagnosed radiologically with a head and neck paraganglioma by magnetic resonance imaging of the brain, which revealed a hypointense and hyperintense punctate mass centered at the jugular fossa with intracranial extension. The ascending pharyngeal artery, recognized as the major feeder, was embolized by percutaneous embolization following digital subtraction angiography. Gross total resection of the tumor was followed by an uneventful postoperative recovery. Combined immunohistochemistry and histopathological morphology revealed a basaloid squamous cell carcinoma, following which the patient completed radiotherapy and is at 3-month follow-up currently.

**Conclusion:**

This case report discusses the diagnostic pitfalls and management challenges of this rare entity on the basis of prior evidence, as well as a literature review and clinical and surgical analysis.

## Introduction

Basaloid squamous cell carcinoma (BSCC) is a rare malignant variant of squamous cell carcinoma (SCC) that usually arises in the neck [1]. We report the case of a patient who was diagnosed radiologically with head and neck paraganglioma (HNPGL). However, subsequent histopathology and immunohistochemistry revealed BSCC. This case highlights the importance of histological confirmation for accurate diagnosis and management.

## Case report

A 66-year-old previously healthy Sinhalese male with no history of tobacco/alcohol use or unprotected sexual activity presented with right-sided hearing impairment associated with vertigo for 6 months. There was no complaint of pulsatile tinnitus, headache, dysphagia, dysarthria, visual disturbances, seizures, or difficulty walking. He had a deviation of the mouth toward the left side, with incomplete closure of the right eye (House–Brackmann grade 2). On examination, right-sided 7th, 8th, 9th, 10th, and 12th cranial nerve palsies were observed. However, cough and gag reflexes were unimpaired, and taste sensation was intact. No masses or lymphadenopathy were observed.

**Fig. 1:**
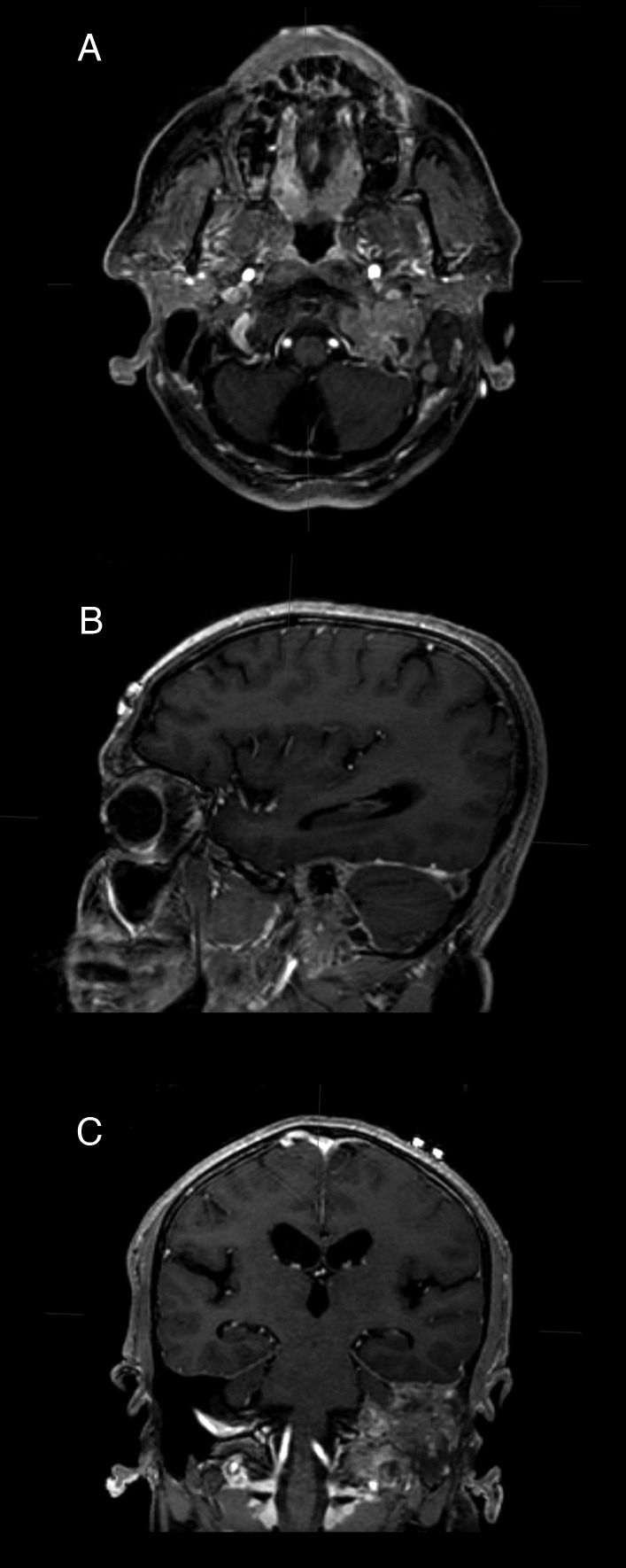
**a** Axial, **b** sagittal, and **c** coronal T1-weighted magnetic resonance images of the tumor revealing a lobulated, rather well-demarcated isointense to hyperintense mass centered in the right jugular fossa infiltrating into the posterior fossa and temporal bone

Contrast-enhanced computed tomography (CECT) of the brain and petrous temporal bone revealed a right middle ear and mastoid air cells filled with soft tissue material and widened, eroded, and filled the right jugular foramen with multiple vascular channels alongside erosion of the right caraticojugular spine. Non-contrast and contrast-enhanced magnetic resonance imaging (MRI) of the brain revealed a lobulated, rather well-demarcated isointense to hyperintense mass centered in the right jugular fossa with intracranial extension infiltrating into the right temporal lobe, extending to the odontoid and mastoid inferiorly and laterally, respectively. The mass displayed hyperintense and hypointense punctuate signals and was enhanced with contrast, suggesting a HNPGL. The mass appeared to have infiltrated the sigmoid sinus while partially encasing the narrowed petrous portion of the internal carotid artery. Head, neck, chest, and abdominal evaluation suggested no evidence of distant metastasis or possible primary tumor (Fig. 1).

Digital subtraction angiography (DSA) conducted on the day of surgery revealed a dominant abnormal blush in the right ascending pharyngeal artery. Selective catheterization and embolization with 300–500 μm polyvinyl alcohol (PVA) particles resulted in complete occlusion of the vessel.

Surgery to excise the right glomus tumor was conducted on the same day as the preoperative embolization. A lateral presigmoid retrosigmoid extracranial skull base approach was used, where a right-sided “?” mark incision was made extending from the anterior margin of the sternocleidomastoid muscle (SCM) behind the ear extending to the temporal region. The SCM was dissected from the anterior border to find the internal jugular vein (IJV) and carotid artery. The muscle was dissected from the mastoid tip, followed by mastoidectomy. The external ear was closed, and the malleus, incus, and ear drum were removed. The pre- and retro-sigmoid bones were drilled, exposing the sigmoid sinus. The tumor was found to extend along the IJV, and a gross total excision of the tumor was performed with hemostasis and preservation of the facial nerve.

**Fig. 2:**
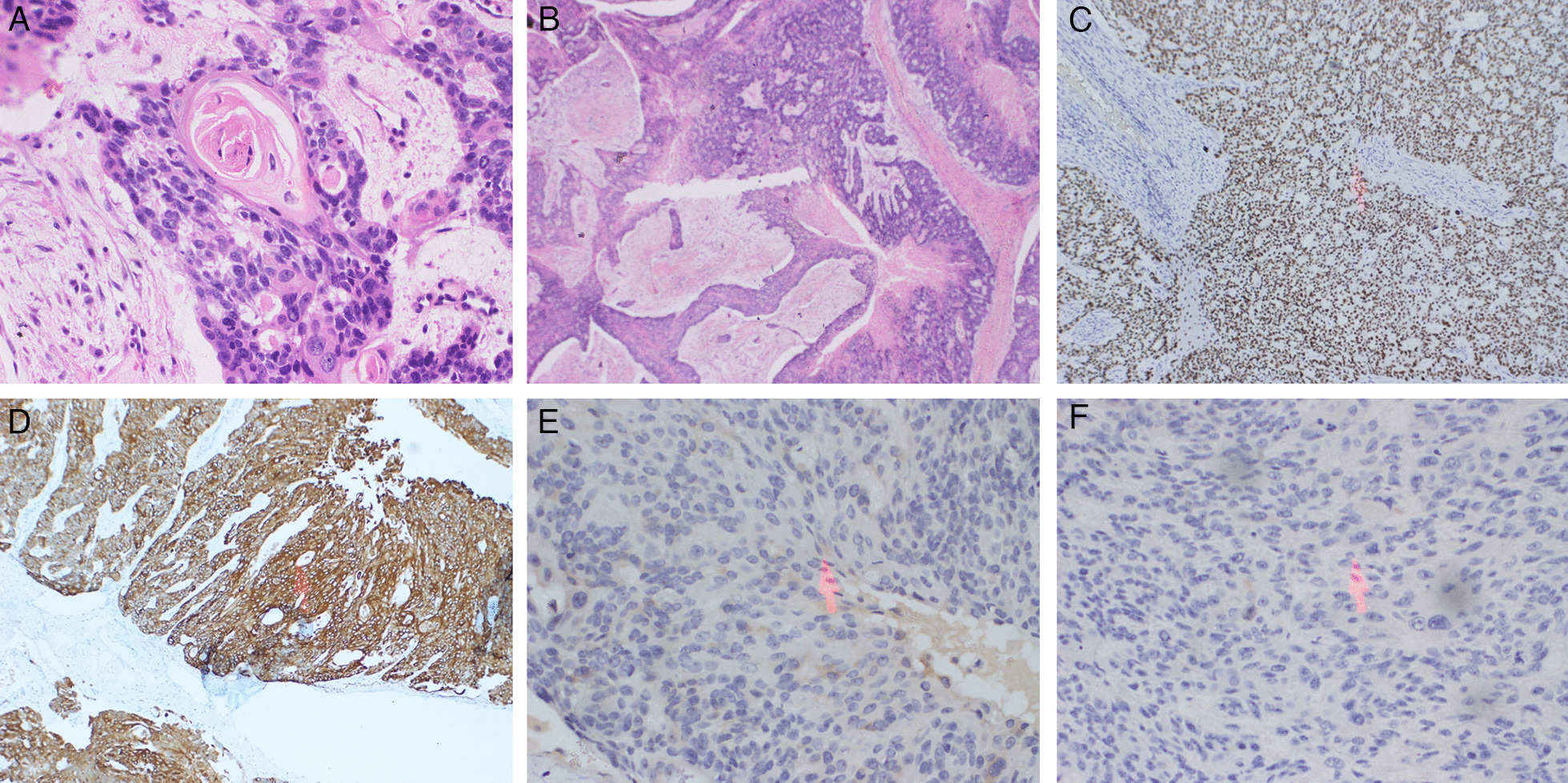
**a**, **b** Hematoxylin and eosin staining of tumor cells demonstrating (**a**) keratin squamous pearls (× 100), (**b**) adenoid cystic (× 10) patterns of neoplastic cells, **c** immunohistochemistry demonstrating strong nuclear positive P63 staining in most of the cells (× 100), **d** immunohistochemistry demonstrating strong diffuse membrane/cytoplasmic positive tumor cells with Ck 5/6 staining (× 100), **e, f** immunohistochemistry demonstrating (**e**) CD117-negative, (**f**) Neuron-Specific Enolase-negative tumor cells after positive staining with Ck 5/6 and P63 (× 10). (Arrowheads in images **c**–**f** demonstrate focus areas of interest in each image)

Tumor sections revealed infiltrative tumors composed of neoplastic cells with solid, trabecular, and cribriform growth patterns. An adenoid cystic-like pattern and rosetting, such as acellular central canals, a small broad perivascular papillary pattern, and squamous metaplasia, were observed. Perineural, bony, and soft tissue infiltration was observed. On immunohistochemistry, positive cytoplasmic and membrane staining with Ck 5/6 and strong nuclear staining with P63 were noted, suggestive of a carcinoma of squamous, basaloid, and adenoid cystic morphology. P16 staining was not performed. Therefore, a combined morphological and immunohistochemistry diagnosis of BSCC was made [2] (Fig. 2).

**Fig. 3:**
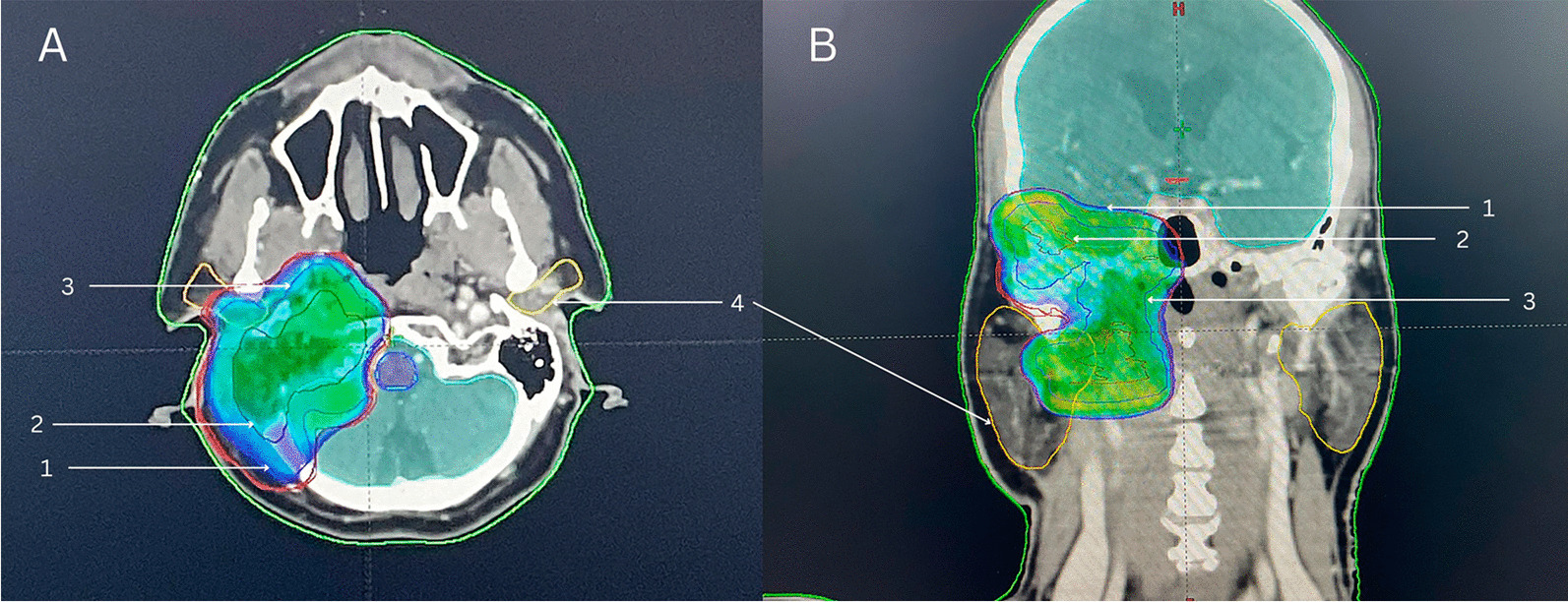
**a** Axial, **b** coronal, image color wash (95% dose) showing the planning target volume (thick blue line; arrow 1). Radical radiotherapy was delivered in the form of intensity modulated radiotherapy targeted to the tumor bed where planning target volume was based on the gross tumor volume (red line; arrow 2), which was delineated on the axial images taken in the treatment position with an immobilization device (thermoplastic mask) with the support of magnetic resonance imaging fusion. The clinical target volume (thin blue line; arrow 3) was taken after providing a margin to the gross tumor volume to cover possible microscopic disease adequately with the inclusion of neural exit foramina to cover the perineural tumor. The clinical target volume was expanded isotopically to form the planning target volume (thick blue line; arrow 1), adding a margin to account for uncertainties. The brainstem, spinal cord, left cochlea and both parotids and lenses were identified as organs at risk (yellow lines; arrow 4), and exposure was kept under the tolerance level.

Postsurgical radical radiotherapy (adjuvant radiotherapy) was planned at a dose of 65 Gy to be delivered in 30 fractions according to the type/location of the tumor and the postsurgical residual tumor volume (Fig. 3).

Currently, the patient has completed all 30 sessions with good compliance and satisfaction and is pending to undergo a 3-month postradiotherapy MRI scan. The patient currently suffers from ipsilateral facial nerve palsy and a hearing deficit equivalent to the preoperative deficit. Radiotherapy-related acute side effects such as nausea, lassitude, malaise, lethargy, focal alopecia, erythema, desquamation, and mucositis are currently being controlled medically by means of analgesics and steroids.

## Discussion

Jugular foramen lesions commonly include paragangliomas followed by certain other types, such as meningiomas, schwannomas, or even chondrosarcomas. Head and neck paragangliomas (HNPGLs) are rare tumors with an estimated clinical incidence of 1/100,000 patients per year. The classical main types of HNPGLs are carotid body tumors, jugular (considered glomus jugulare tumors or GJTs in the current context) and tympanic paranganglial tumors, which are collectively considered JTPGLs and vagal paraganglial tumors [3]. Furthermore, metastatic localization within the region of the jugular foramen is rare, accounting for approximately 3.5–36% of all secondary malignant tumors involving the temporal bone [4].

GJTs are the most common form of paragangliomas and hence the most common of all jugular foramen tumors. They are highly vascularized histologically benign lesions known to arise from the temporal bone paraganglial system. However, these tumors are known to be locally invasive by invading adjacent bone, blood vessels, and the central nervous system [5]. In contrast, BSCC is a more aggressive rare form of SCC (accounting for 5% of all SCCs) with a greater potential for distant metastasis and poor prognosis [6].

Considering demographic variations, HNPGLs are commonly observed in women in their late 40s, while BSCCs are usually common in men in their 60s with a history of tobacco/alcohol use [7, 8]. A retrospective analysis of 20 patients diagnosed with temporal bone metastases revealed a median age of detection of 60 years, with a majority being men (13 out of 20). This finding highlights the importance of considering metastatic lesions in male patients of advanced age despite presenting with classical features of GJ tumors [9].

The clinical presentations of JTPGLs usually involve pulsatile tinnitus, ear mass, hearing loss, pain, and vertigo attributed to mass effects dominated by the involvement of cranial nerves. Hence, a preoperative cranial nerve defect is frequently observed, especially in JTPGLs, rather than in other HNPGLs [8]. Furthermore, temporal bone metastases are usually associated with otological symptoms of hearing loss in addition to other neurological manifestations, such as facial nerve palsy, while the initial presentation of BSCCs usually includes neck lymph node metastases [1, 9]. This finding is similar to that of other known cases of temporal bone metastases presenting as glomus jugulare tumors as well [10–13]. However, it is important to note that lower cranial nerve palsies involving the IX^th^, X^th^, XI^th^ (and XII^th^) cranial nerves (CNs), which are seen in HNPGLs because of mass effects, are also considered common features of metastatic lesions, described in the literature as Jugular Foramen (JF) Vernet syndrome (involving IX^th^, X^th^, and XI^th^ CNs), posterior laterocondylar syndrome (Vernet plus XII^th^ CN), and posterior retropharyngeal syndrome (Vernet plus XII^th^ and Horner’s) [12]. Likewise, a 1976 review of temporal bone metastases also described a triad of facial paralysis, otalgia, and periauricular swelling to alert a clinician of metastatic temporal bone disease [14]. While classical features of JTPGLs, such as hearing loss, tinnitus, and vertigo, were observed in this patient, the presence of the combination of VII^th^ and lower cranial nerve palsies highlights the importance of cranial nerve palsies dominating otological symptoms in the exclusion of metastatic lesions and that the mere absence of the classical triad or the various JF syndromes are not reliable indicators of benign lesions.

Both biochemical and radiological diagnostic tests play major roles in overcoming dilemmas associated with complex presentations. This is especially relevant in cases of JTPGLs since diagnostic biopsies are discouraged or even contraindicated due to the risk of precipitating hypertensive crisis, hemorrhage, or tumor seeding [3]. Radiological investigations include CT and MRI as first-line methods, where CT scans, though less sensitive, accurately define possible bone invasion and MRIs display a characteristic salt-and-pepper appearance [3]. An analysis of 236 patients with benign paragangliomas revealed that 26 of the 27 patients with GJTs who underwent CT scans were true positives, while all 24 patients with GJTs who underwent MRI were true positives, illustrating the paramount importance of imaging in patients with clinical suspicion of HNPGLs [8]. On the contrary, biochemical studies, especially those involving plasma or 24-hour urinary metanephrine or catecholamine concentrations, can be effectively used in certain instances since these tumors can rarely be secretory. This is evident from the higher sensitivities of 24-hour urine studies observed in the same analysis, which revealed a combined sensitivity of 89.9% [8]. CTs and MRIs were both used in this case to arrive at the diagnosis, where the hyperintense and hypointense punctate signals in preoperative MRI corresponding to the classical appearance proved to be wrong. Endocrine studies were not conducted due to pragmatic difficulties. Hence, it is important to note that while classical appearances are highly useful in diagnosis, metastatic lesions should nevertheless always be considered, and scans involving the neck, chest, and abdomen ought to be carried out as well to exclude potential primaries that might have led to a masquerading metastasis.

Angiography [digital subtraction angiography (DSA) or MRI angiography] is also an important modality not only for diagnosing and defining vascular anatomy preoperatively, but also for embolizing major feeders where it is recommended for the management of resectable lesions. However, angiography and embolization can play more pivotal role in reducing intraoperative complications and facilitate surgical radicality over curing or reducing clinical sequelae [15]. A review of 38 patients with PGL at the Mayo Clinic revealed that preoperative embolization was associated with a 75% average reduction in blood flow to the tumor, emphasizing its value in reducing intraoperative bleeding. As in this case, the ascending pharyngeal branch of the external carotid artery was the most common major feeder for PGLs (8 GJTs), thus indicating its minimal or nil role in diagnostics [16].

Surgery for the management of JTPGLs is the most common treatment and is a rather challenging task because of the need to explore the posterolateral skull base. This liniency is evident by the 71% majority of the 67 GJTs that underwent surgery in the retrospective chart review of patients with JF tumors. Despite complexities associated with surgery, gross total resection is common in many cases (59–69%), with an associated mortality rate of less than 2% [3]. However, the functional outcome following surgery is rather poor, with long-term facial nerve dysfunction in 14–33% of cases; audiological deterioration in as much as 39% of the cases; and a global risk of lower CN deficits ranging from 18% to 40%, which was not applicable in the present case, where there was no significant functional decline [17, 18]. Hence, careful preoperative and intraoperative analysis of tumor extensions should be combined with expert surgical techniques to ensure complete resection of the tumor while preserving vital neurovascular structures as much as possible and ensuring minimal potential recurrence and seeding.

Finally, the use of radiotherapy for the postsurgical management of BSCCs of the temporal bone, such as in this case, should be decided after careful evaluation of Human Papilloma Virus (HPV) status, perineural extension, and the presence of positive or close margins via diagnostic modalities such as MRI while ensuring that the primary tumor bed is covered. Intensity-modulated radiotherapy is considered to be highly effective in the management of SCCs, and surgery and chemoradiotherapy are considered to be best suited for this purpose according to a multitude of case‒control studies [19]. Surgical sampling is always beneficial in tailoring the treatment regimen for these patients since tumor debulking and histopathological confirmation reduce the tumor load and the type of tumor to target [1].

## Conclusion

Hence, both the present case and the literature are consistent in portraying clinical symptoms as the best delineating feature to be used in identifying metastatic lesions from JTPGLs. Therefore, we suggest that male sex, advanced age, facial nerve palsy, and/or variable lower CN palsy should be given emphasis in ruling out metastatic lesions despite the presence of classical audiological and radiological features suggestive of JTPGLs. Furthermore, postoperative radiotherapy should be started only once a combined morphological and immunohistochemical diagnosis is made to ensure effective treatment. We recommend further analysis to evaluate the validity of the application of these hypotheses in wider patient populations.

This first case of BSCC masquerading as an HNPGL highlights that malignant tumors can create diagnostic dilemmas and hence require careful evaluation and exclusion. Furthermore, surgical experience and careful planning are essential to ensure favorable outcomes in these patients.

## Data Availability

All patient records, operation notes, and radiographic information are available in the form of hard copies. Scanned documents can be provided upon request from the journal.

## References

[1] Zbären P, Nuyens M, Stauffer E (2004). Basaloid squamous cell carcinoma of the head and neck. Curr Opin Otolaryngol Head Neck Surg.

[2] El-Naggar AK, Chan JKC, Grandis JR, Takata T, Slootweg PJ. Mucoepidermoid Carcinoma. In: WHO Classification of Head and Neck Tumours. WHO classification of Head and neck Tumours, 2017. 10.1016/j.humpath.2017.05.014.

[3] Capatina C, Ntali G, Karavitaki N, Grossman AB (2013). The management of head-and-neck paragangliomas. Endocr Relat Cancer.

[4] Ciavarro G, Bozzetti F, Falcioni M (2019). Jugular foramen metastasis from lung cancer: a case of “a mass without his syndrome”. J Int Adv Otol.

[5] Fayad JN, Keles B, Brackmann DE (2010). Jugular foramen tumours: clinical characteristics and treatment outcomes. Otol Neurotol.

[6] Wain SL, Kier R, Vollmer RT, Bossen EH (1986). Basaloid-squamous carcinoma of the tongue, hypopharynx, and larynx: report of 10 cases. Hum Pathol.

[7] Barnes L, Macmillan C, Ferlito A, Rinaldo A, Altavilla G, Doglioni C (1996). Basaloid squamous cell carcinoma of the head and neck: clinicopathological features and differential diagnosis. Ann Otol Rhinol Laryngol.

[8] Erickson D, Kudva YC, Ebersold MJ (2001). Benign paragangliomas: clinical presentation and treatment outcomes in 236 patients. J Clin Endocrinol Metab.

[9] Song K, Park KW, Heo JH, Song IC, Park YH, Choi JW (2019). Clinical characteristics of temporal bone metastases. Clin Exp Otorhinolaryngol..

[10] Hellier WPL, Crockard HA, Cheesman AD (1997). Metastatic carcinoma of the temporal bone presenting as glomus jugulare and glomus tympanicum tumours: a description of two cases. J Laryngol Otol.

[11] Das AK, Singh SK, Bhavana K, Kumar S (2023). Posterior fossa giant adenoid cystic carcinoma with skull base invasion mimicking glomus jugulare: a case report and review of literature. Rare Tumours.

[12] Boileau MA, Grotta JC, Borit A (1987). Metastatic renal cell carcinoma simulating glumus jugulare tumour. J Surg Oncol.

[13] Thomas AJ, Wiggins RH, Gurgel RK (2017). Metastatic renal cell carcinoma masquerading as jugular foramen paraganglioma: a role for novel magnetic resonance imaging. Ann Otol Rhinol Laryngol.

[14] Maddox HE (1967). XI. Metastatic tumours of the temporal bone. Ann Otol Rhinol Laryngol.

[15] Tasar M, Yetiser S (2004). Glomus tumours: therapeutic role of selective embolization. J Craniofac Surg.

[16] White JB, Link MJ, Cloft HJ (2008). Endovascular embolization of paragangliomas: a safe adjuvant to treatment. J Vasc Interv Neurol.

[17] Briner HR, Linder TE, Pauw B, Fisch U (1999). Long-term results of surgery for temporal bone paragangliomas. Laryngoscope.

[18] Ivan ME, Sughrue ME, Clark AJ (2011). A meta-analysis of tumour control rates and treatment-related morbidity for patients with glomus jugulare tumours. J Neurosurg.

[19] Yao M, Dornfeld KJ, Buatti JM (2005). Intensity-modulated radiation treatment for head-and-neck squamous cell carcinoma—The University of Iowa experience. Int J Radiat Oncol Biol Phys.

